# Gut Microbiota Interacts with Dietary Habits in Screenings for Early Detection of Colorectal Cancer

**DOI:** 10.3390/nu17010084

**Published:** 2024-12-28

**Authors:** Ana Vega-Rojas, Carmen Haro, Helena Molina-Abril, Silvia Guil-Luna, Jose Antonio Santos-Marcos, Francisco Miguel Gutierrez-Mariscal, Helena Garcia-Fernandez, Javier Caballero-Villarraso, Antonio Rodriguez-Ariza, Jose Lopez-Miranda, Pablo Perez-Martinez, Antonio Hervas, Antonio Camargo

**Affiliations:** 1 Lipids and Atherosclerosis Unit, Department of Internal Medicine, Reina Sofia University Hospital, 14004 Cordoba, Spain; anavero.90@gmail.com (A.V.-R.); santosmarcosjoseantonio@gmail.com (J.A.S.-M.); francisco.gutierrez@imibic.org (F.M.G.-M.); helena.garcia@imibic.org (H.G.-F.); jlopezmir@uco.es (J.L.-M.); 2 Department of Medical and Surgical Sciences, University of Cordoba, 14004 Cordoba, Spain; 3 Maimonides Institute for Biomedical Research in Cordoba (IMIBIC), 14004 Cordoba, Spain; v22gulus@uco.es (S.G.-L.); bc2cavij@uco.es (J.C.-V.); antonio.rodriguez.exts@juntadeandalucia.es (A.R.-A.); 4 CIBER Fisiopatologia de la Obesidad y Nutricion (CIBEROBN), Instituto de Salud Carlos III, 28029 Madrid, Spain; 5 Institute for Sustainable Agriculture, Spanish National Research Council (IAS-CSIC), 14004 Cordoba, Spain; charo@ias.csic.es; 6 Department of Applied Mathematics I, University of Seville, 41012 Seville, Spain; hmabril@gmail.com; 7 Cancer Network Biomedical Research Center (CIBERONC), 28029 Madrid, Spain; 8 Department of Anatomy and Comparative Pathology and Toxicology, University of Cordoba, 14004 Cordoba, Spain; 9 Department of Biochemistry and Molecular Biology, University of Cordoba, 14004 Cordoba, Spain; 10 Medical Oncology Department, Reina Sofia University Hospital, 14004 Cordoba, Spain; 11 Digestive Department, Reina Sofia University Hospital, 14004 Cordoba, Spain; ahervasm@live.com

**Keywords:** gut microbiome, dietary habits, colorectal cancer, screening program, early diagnosis, random forest

## Abstract

Background/Objectives: Gut microbiota interacts with nutrients, which may be relevant to assigning a microbial signature to colorectal cancer (CRC). We aim to evaluate the potential of gut microbiota combined with dietary habits in the early detection of pathological findings related to CRC in the course of a screening program. Methodology: The colonoscopy performed on 152 subjects positive for fecal occult blood test showed that 6 subjects had adenocarcinoma, 123 had polyps, and 23 subjects had no pathological findings. Gut microbiota was analyzed by 16S metagenomic. Caret package was used to build the classification models in R. Results: Random forest (RF) classifier models were used to test the potential of gut microbiota alone or combined with dietary habits as a biomarker to discern between individuals with CRC-related lesions (polyps or adenocarcinoma) versus individuals without pathological findings. RF classifier models yielded an area under the curve of 0.790 using gut microbiota data, 0.710 using dietary habits data, and 0.804 in the combined model including gut microbiota and dietary habits data. The abundance of *Suterella*, *Oscillospirales*, *Proteobacteria*, and *Burkholderiales* was highly discriminant between groups, together with the consumption of fruit and vegetables and the consumption of carbonated and/or sweetened beverages. Conclusions: Our results suggest that the interaction between gut microbiota and dietary habits is relevant when a microbial signature is used as a marker in CRC. Moreover, gut microbiota signature and information about the dietary habits of the individuals seem to be important for improving screening programs for the early detection of CRC.

## 1. Introduction

Colorectal cancer (CRC) is the third most common cancer worldwide, accounting for approximately 10% of all cancer cases and with an incidence that continues to increase in the population [1,2]. In addition, CRC remains latent for years before it is diagnosed. The incidence and impact of CRC can be significantly reduced by implementing screening programs in the at-risk population to enable early detection and subsequently to improve patient outcomes [3].

Current clinical diagnostic procedures, such as the fecal occult blood test (FOBT), have limited specificity for detecting CRC [4]. In addition, colonoscopy methods pose limitations, such as invasiveness, patient discomfort, and resource constraints, thereby making it necessary to improve the methods for CRC screening programs [5,6].

The identification of novel biomarkers, which are reliable and non-invasive, is extremely useful in the design of non-invasive tools for early CRC diagnosis. It has been estimated that the use of accurate tests for screening average-risk individuals can reduce the incidence and mortality associated with CRC [7].

The gut microbiota is currently considered as an organ fully integrated in the host metabolism. Indeed, conditions of microbiota imbalance, also called dysbiosis, are associated with the development of human diseases [8,9,10]. In line with this, research over the past decade has established that dysbiosis of gut bacteria accompanies CRC, and these changes may be causative [11,12].

Moreover, lifestyle factors such as a sedentary lifestyle, smoking, excessive alcohol consumption, and low intake of fruits and vegetables contribute to the development of CRC [13]. In turn, diet is one of the factors which most extensively influences gut microbiota structure and composition [14,15]. Therefore, when gut microbiota is analyzed due to its importance in the development of CRC, the potential interaction between gut microbiota and nutrients should be taken into account.

Herein, we explored the potential interaction between dietary habits and the intestinal microbiota by a random forest approach to discern among individuals with different CRC-related pathological findings. In this study, we aim to evaluate the potential of gut microbiota combined with dietary habits in the early detection of pathological findings related to CRC in the course of a screening program.

## 2. Materials and Methods

### 2.1. Study Population

The CCR-microbiota Study (Gut Microbiota and Color-rectal Cancer; Clinical Trials.gov.Identifier: NCT04662853) is a prospective trial of 153 patients positive in the fecal occult blood test (FOBT) to whom a study by colonoscopy was performed and their gut microbiota characterized by 16S metagenomic. One patient was discarded because of low sequencing quality (*n* = 152). This study was carried out in the framework of the Program for Early Detection of Colon and Rectal Cancer, undertaken by the Consejeria de Salud de la Junta de Andalucia (Spain), which screens the Andalusian population aged between 50 and 69 years old for CRC presence, first by FOBT and later by colonoscopy on the subjects positive for this test. Subjects with positive results in the FOBT were invited to participate in the CCR-microbiome study and were recruited between January 2017 and March 2020, at the Reina Sofia University Hospital (Cordoba, Spain), with the consumption of antibiotics within the previous month as exclusion criteria. The fecal sample was collected a week before colonoscopy to avoid potential effects from the specific diet recommended to the subjects to consume in the 3 days preceding the colonoscopy. In addition, the usual dietary habits were assessed by a validated 14-item questionnaire for all enrolled patients [16]. All patients gave their written informed consent to participate in this study. The trial protocol and all amendments were approved by the Reina Sofia (Cordoba) University Hospital Ethics and Research Committees, following the Helsinki declaration and good clinical practices.

### 2.2. Fecal Occult Blood Test Subsection

The OC-SENSOR Faecal Immunochemical Test was used for quantitative measurement of hemoglobin in feces for colorectal cancer screening. Participants were contacted by phone, and a test was sent by mail to those who accepted being included in the screening program. It was returned by participants to the closest health center, from which it was sent to Reina Sofia University Hospital to be analyzed. A threshold of 20 μgr hemoglobin per gr of feces was used.

### 2.3. Dietary Habits Assessment

The usual dietary habits were assessed by a 14-item questionnaire to assess the adherence to the Mediterranean diet of the participant in this study (MEDAS score) [16].

### 2.4. Colonoscopy and Clinicopathological Data

Subjects positive for FOBT were referred to hospital for colonoscopy. They were asked to avoid fiber-rich diet in the 3 preceding days to the colonoscopy in addition to the ingestion of 2 doses of Macrogol 3350 (Moviprep^®^) separated by 10–12 h, with the second dose 5 h before the colonoscopy appointment time. Colonoscopies were performed with an Olimpus 180/190 series gastrointestinal endoscopy and Olimpus 190 series video processor by qualified medical doctors from the Digestive Service of Reina Sofia University Hospital. Resections of lesions were carried out with polypectomy loops of various diameters, with cold or hot cut or high-capacity biopsy forceps, depending on the size of the polyp. Clinicopathological data were collected for each patient. A centralized study of the histological samples was carried out in the Pathological Anatomy Service of Reina Sofia University Hospital. Following colonoscopic examination, patients without pathological findings were designated as healthy (control group). Examinations that revealed the presence of colonic lesions were submitted for histopathological evaluation and subsequent diagnosis. Colonic samples in which at least one polyp was identified were included in the polyp group, and if at least one adenocarcinoma was diagnosed, they were included in the adenocarcinoma group.

### 2.5. Intestinal Microbiota Analysis

DNA extraction from feces was performed using the QIAamp DNAStool Mini Kit Handbook (QIAGEN, Hilden, Germany), following the manufacturer’s instructions. The microbiota composition analysis of the fecal samples was performed on a MiSeq Illumina platform (Illumina, San Diego, CA, USA), as previously described [17]. Briefly, sequencing data were analyzed and visualized using QIIME2 [18], using the DADA2 method [19]. We evaluated the bacterial alpha [20] and beta diversity [21], the latter analyzed by permutational multivariate analysis of variance (PERMANOVA). Taxonomy was assigned to the high-quality reads using q2-feature-classifier [22] with a sequence identity threshold of 99% interrogating the sequences with the SILVA database [23]. To exclude bacterial taxa that were not present in the majority of samples, a cut-off for exclusion was fixed; only bacterial taxa containing sequence reads in at least 75% of total samples were considered [24]. Linear discriminant analysis (LDA) effect size (LEfSe) was used to compare groups at baseline and visualize the results using taxonomic bar charts and cladograms [25].

### 2.6. Data Modeling

We used random forest (RF) classifier [26] to identify the potential of gut microbiota as biomarker to discern between individuals with CRC-related lesions versus individuals without pathological findings. The combination of gut microbiome and dietary habits was also considered. Caret R package version 6.0–94 was used to build the classification models in R version 4.2.3. Patients with adenocarcinoma and with polyps were grouped together within the same class and compared to subjects without pathological findings. Parameters of the RF classification models were optimized using grid search, and down-sampling techniques were applied to address for class imbalance. Ten-fold cross-validation was used to test the model’s performance, and mean decrease in accuracy identified variables with greater importance within each model.

### 2.7. Statistical Analysis

PASW statistical software package, version 20.0 (IBM Inc., Chicago, IL, USA), was used for statistical analysis of the data. Chi-squared test was used to test differences in dietary habits between groups of subjects. *p* < 0.05 was considered statistically significant. Data of age and BMI are presented as mean ± standard error of the average.

## 3. Results

### 3.1. Characteristic of the Participants in the Study

A total of 152 subjects (92/60 men/women, age 64.63 ± 0.36 y, BMI 29.10 ± 0.34 kg/m^2^) were included in this study. Among these patients, 6 had adenocarcinoma (adenocarcinoma group, 6 men, aged 63.33 ± 2.36 y, BMI 25.87 ± 0.73 kg/m^2^), 123 had polyps (polyp group, 75/48 men/women, age 64.93 ± 0.37 y, BMI 29.52 ± 0.37 kg/m^2^), and 23 subjects had no pathological findings (control group, 11/12 men/women, aged 63.35 ± 1.17 y, BMI 27.71 ± 0.87 kg/m^2^).

### 3.2. Dietary Habits Assessment of the Participants in This Study

**Figure 1:**
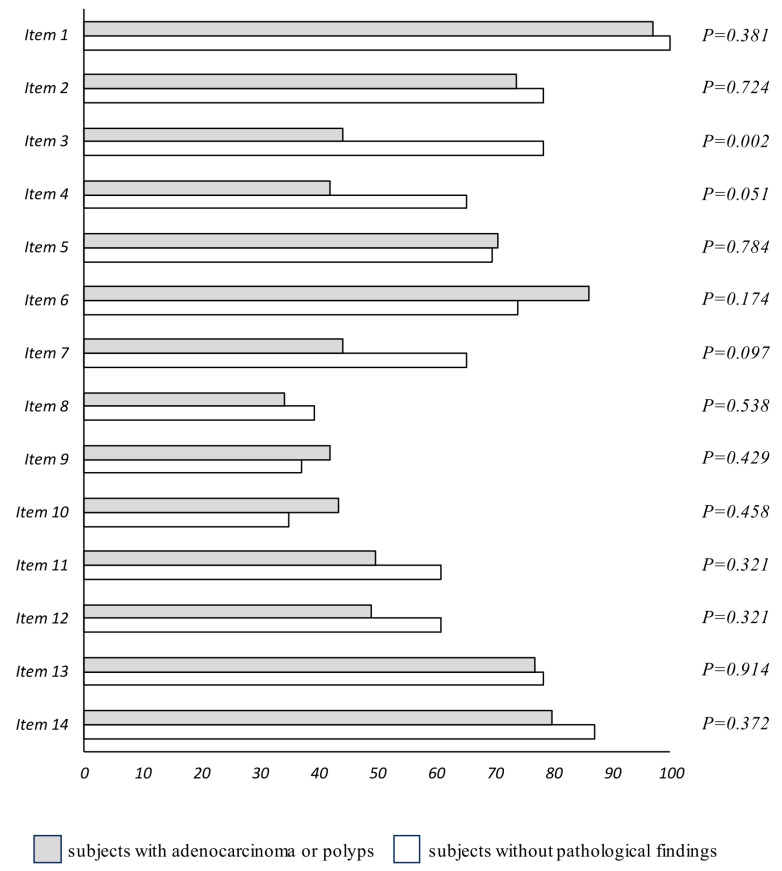
Dietary habits assessment based on different items of the Mediterranean diet adherence questionnaire. The usual dietary habits were assessed by a validated 14-item questionnaire [16]. Differences between the compliance of the different items were analyzed by chi-squared test.

In order to assess the adherence to the Mediterranean diet of the participant in this study, a 14-item MEDAS score was used. While we did not find significant differences in global adherence to the Mediterranean diet between groups, we found a different compliance according to the item. In fact, we found that there was a higher compliance to item 3 (high vegetable consumption), item 4 (high fruit consumption), and item 7 (low carbonated and/or sweetened beverages consumption) in subjects without pathological findings (Figure 1).

### 3.3. Diversity of the Gut Microbiota According to the Presence of CRC-Related Pathological Findings

We analyzed alpha diversity as assessed by Shannon, Simpson (1-D), Chao1, and Faith’s phylogenetic diversity indices. We observed a statistically significant difference in alpha diversity (*p*-values = 0.004, 0.006, 0.008, and 0.042, respectively). Further Kruskal–Wallis pairwise analysis showed lower alpha diversity in patients included in polyp groups as compared with the group of subjects without pathological findings (*p*-values 0.001, 0.001, 0.003, and 0.013, respectively).

Moreover, we also found significant distinctions in beta diversity among subjects classified in the adenocarcinoma group, polyp group, and the group of subjects without pathological findings, which held true across Jaccard (*p*-value = 0.001) and Bray–Curtis (*p*-value = 0.005) distances, qualitative and quantitative measures, respectively. When considering bacterial phylogeny, we did not find significant distinctions in unweighted and weighted Unifrac distances (*p*-value = 0.072 and *p*-value = 0.186, qualitative and quantitative, considering bacterial phylogeny, respectively).

### 3.4. Differences in the Gut Microbiota According to the Presence of CRC-Related Pathological Findings: LEfSe Analysis

**Figure 2:**
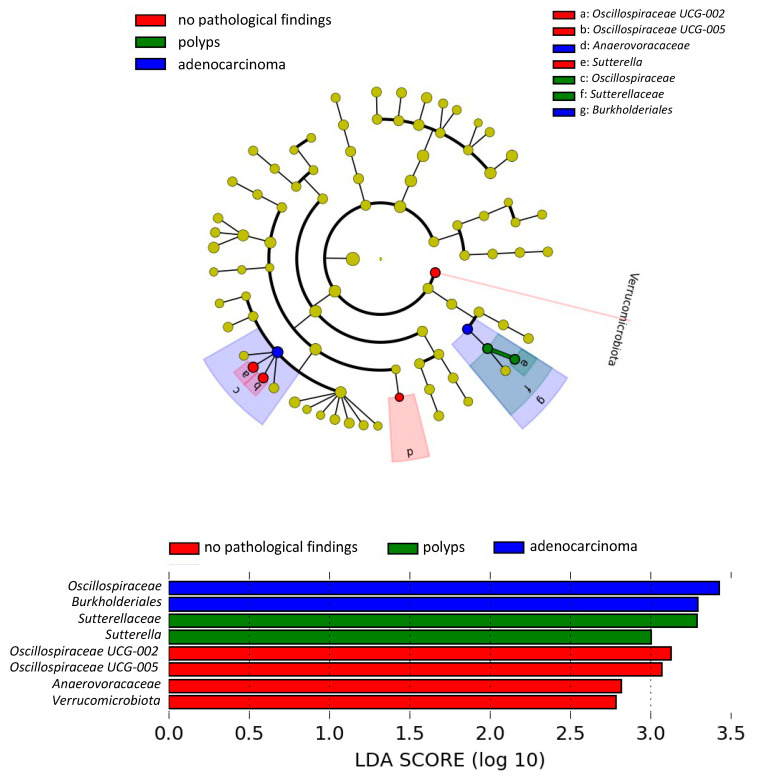
Differently abundant taxa identified using LEfSe analysis. The most differently abundant taxa between the groups under study are represented in a bar graph according to the LDA score (log 10), in an estimation of the effect size, and in a taxonomic cladogram. Only taxa meeting a *p* < 0.05 and LDA score significant threshold |>2| are shown. The colors represent the group in which the indicated taxa are more abundant compared to the other groups. In a taxonomic cladogram, each successive circle represents a different phylogenetic level. The order from the center to the outside is phylum, class, family, and genus levels.

We employed LEfSe to identify taxonomic variations between subjects with adenocarcinoma, subjects with polyps, and subjects with no pathological findings (Figure 2). The gut microbiota of subjects with adenocarcinoma was enriched in *Oscillospiraceae* family and *Burkholderiales* order, the gut microbiota of subjects with polyps was enriched in *Sutterellaceae* family and *Sutterella* genus, and the gut microbiota of subjects with no pathological findings was enriched in *Verrucomicrobiota* phylum, *Anaerovoracaceae* family, and *Oscillospiraceae UCG-005* and *Oscillospiraceae UCG-002* genera.

### 3.5. Random Forest Classifier Analysis

We performed RF classifier models to test the potential of gut microbiota alone or combined with dietary habits as a biomarker to discern between individuals with CRC-related lesions (polyps or adenocarcinoma) versus individuals without pathological findings. Several RF classifier models were created based on the following data: (1) the microbiome (bacterial composition expressed as relative abundance); (2) dietary habits based on the Mediterranean diet compliance questionnaire analyzing the 14 items together as a measure of the global adherence to the Mediterranean diet; (3) the microbiome combined with dietary habits in terms of adherence to a diet; (4) the microbiome data combined with dietary habits based on the Mediterranean diet compliance questionnaire analyzing each item separately.

**Figure 3:**
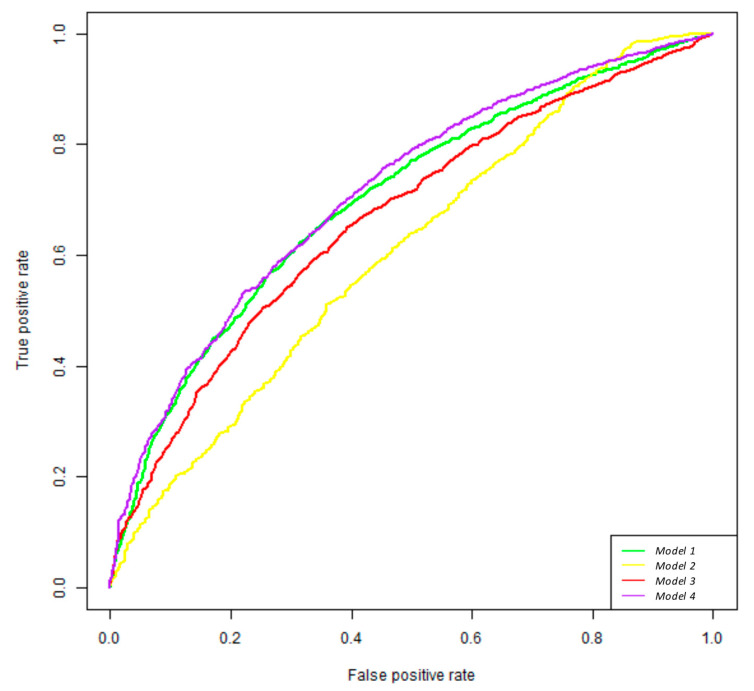
ROC curves built based on the random forest classifier analysis. Several random forest classifier models were created based on the following data: model (1) the microbiome (bacterial composition expressed as relative abundance); model (2) dietary habits based on the Mediterranean diet compliance questionnaire analyzing the 14 items together as a measure of the global adherence to the Mediterranean diet; model (3) the microbiome combined with dietary habits in terms of adherence to a diet; model (4) the microbiome data combined with dietary habits based on the Mediterranean diet compliance questionnaire analyzing each item separately.

The performance of the models on unseen data was tested using 10-fold cross-validation. Error curves and performance measures were calculated using pROC and Caret R packages. The average area under the curve was 0.790 for the model using microbiome data and 0.710 for the one containing dietary habits in terms of items of diet compliance. The combination of microbiome data with dietary habits in terms of adherence to a diet increased performance up to 0.801, and with the dietary habits in terms of items of diet compliance, it increased up to 0.804 (Figure 3 and Figure S1). The predictive value of each variable in the random forest models was calculated using a mean decrease in accuracy. Bacterial taxa with higher importance identified by model 1 are shown in Table 1. Relevant dietary habits in terms of items of diet compliance identified by model 3 are shown in Table 2. All measures of importance were scaled to have a maximum value of 100.

## 4. Discussion

Our study, conducted as part of a screening program in FOBT-positive individuals who underwent colonoscopy, showed that a gut microbiota profile associated with CRC-related lesions was able to distinguish subjects without pathological findings from those with polyps or adenocarcinoma. RF classifier methodology identified the most discriminant bacterial taxa to discern between individuals with polyps or adenocarcinoma and individuals without pathological findings. However, this model did not significantly improve when usual dietary habits were added, as assessed by dietary compliance items. Nevertheless, in this combined model, the two most important discriminant variables were dietary items related to the consumption of fruits and vegetables, followed by several bacterial taxa.

Primary prevention and screening programs for CRC are crucial in contributing to a healthy society and saving lives. Indeed, this research was carried out as part of the screening program of the Andalusian Health Service for the population over 50 years of age. Considering that one of the key factors involved in colorectal carcinogenesis is the environment of the gut microbiome [27], we analyzed the gut microbiota before colonoscopy in patients with positive FOBT results who underwent colonoscopy.

Based on the colonoscopy findings, we classified patients between individuals with polyps or adenocarcinoma and individuals without pathological findings. The discriminant analysis by LefSe software revealed distinct dominant microbial communities unique to each group. In line with this, the intestinal microbiota has previously been proposed as a predictor of the development of human diseases, such as neurological, intestinal, and autoimmune diseases [8,9,10]. Interestingly, research over the past decade has established a causal relationship between intestinal microbial dysbiosis and CRC pathogenesis [11,12].

Our study explored the potential interaction between dietary habits and the intestinal microbiota by an RF approach to distinguish individuals with polyps or adenocarcinoma from those individuals without pathological findings. The use of the RF algorithm to classify between CRC and healthy individuals has recently been reported [28]. Herein, this work showed a better model using the gut microbiota data set than dietary habits, although the gut-microbiota-based model did not significatively improve when adding usual dietary habits assessed by dietary compliance items. The data modeling by an RF approach showed through the combined model a group of bacteria that are highly discriminant between individuals with polyps or adenocarcinoma and those individuals without pathological findings. Indeed, two genera from the *Oscillospirales* order and the *Sutterella* genus were also detected in the previous LefSe analysis. Indeed, the abundance of *Sutterella* genus has been shown to increase in patients with adenocarcinomas [29] and is associated with CRC stage I [30]. Moreover, members of the *Oscillospirales* order have been associated with patients with adenocarcinomas [31]. Surprisingly, the bacterial genus *Fusobacterium nucleatum*, which has been associated with CRC [32], was poorly detected in our population.

Despite the importance of gut microbiota in the development of CRC, the combined model including gut microbiota and dietary habits data showed two variables related to the consumption of vegetables (item 3) and the consumption of carbonated and/or sweetened beverages (item 7) as the two most important discriminant variables in the model. These results suggest the importance of nutrition in CRC development. Indeed, diet plays an important role in the development and progression of many cancers, including CRC [33]. Moreover, diet is one of the major determinants of gut microbiota composition [34], and changes in diet are accompanied by changes in the fecal microbiota [35]. Taken together, our results suggest the importance of the nutritional aspects in screening programs for the early detection of CRC.

Consequently, gut microbiota, which is shaped by lifestyle and dietary habits, may be critically involved in the pathogenesis of CRC even in preneoplastic lesions such as polyps, as shown in this study. Our results suggest that the gut microbiota profile associated with CRC depends on dietary habits and gut microbiota, and both are shifting gut microbiota composition. In addition, the gut microbiota–dietary habits interaction may explain, at least partially, the fact that the gut-microbiota-based model did not significatively improve when adding usual dietary habits.

Our study suggests that a low consumption of fruit and vegetables in addition to a high consumption of carbonated and/or sweetened beverages is associated with the presence of polyps or adenocarcinoma development. In line with this, multiple studies have focused on the relationship between intake of fruit and vegetables and the risk of CRC, showing a reduced risk associated with a high consumption of fruit and vegetables [36,37,38]. In fact, the proposed mechanisms for this negative association are related to antitumor properties by regulating cell signaling and/or proliferation pathways [39].

In the last few years, the relationship between sweetened beverages and the risk of overall cancer [40] has been proposed. Indeed, it has been shown that the consumption of sweetened beverages is associated with the risk of CRC [41,42,43], although the influence of sugar content is still unclear [44]. According to this, it has been proposed that the adverse effects of excess consumption of sweetened beverages may involve alteration of gut microbiota, which would lead to the development of CRC. However, the research available is scarce, and the understanding of the extent to which excessive consumption of sweetened beverages can alter the gut microbiota is limited [45].

Our study has limitations. Diet is one of the most important lifestyle factors in shaping gut microbiota, and this study was limited to analyzing the interaction between the gut microbiota and the development of CRC, not including other lifestyle factors such as smoking, sedentarism, or alcohol consumption. Another limitation was the sample size. However, due to the limited number of samples, a cross-validation strategy was used to evaluate the proposed models.

## 5. Conclusions

Our results suggest that the interaction between the gut microbiota and dietary habits is relevant when microbial signatures are used as a marker in colorectal cancer. Moreover, gut microbiota structure and composition and information about the dietary habits of the individuals seem to be important in order to improve screening programs for the early detection of CRC.

## Figures and Tables

**Table 1:** Importance of the variables in model 1. This model was built including microbiome data (bacterial composition expressed as abundance) to discern between individuals with CRC-related lesions (polyps or adenocarcinoma) versus individuals without pathological findings.

20 More Important Variables in the Model 1	*Importance*
*Ruminococcaceae* family	100.00
*Collinsella* genus	58.21
*Faecalibacterium* genus	54.12
*Anaerovoracaceae* family	52.08
*Streptococcus* genus	47.10
*Negativibacillus* genus	42.81
*Barnesiella* genus	41.27
*Streptococcaceae* family	35.99
*Veillonellaceae* family	35.59
*Verrucomicrobiota* phylum	31.90
*Gammaproteobacteria* class	31.77
*Burkholderiales* order	24.83
*Oscillospirales* order	24.16
unknown genus from *Lachnospiraceae* family	20.54
*NK4A214* group (*Oscillospiraceae* family)	20.21
*Coriobacteriales* order	20.12
*UCG-005* genus (*Oscillospiraceae* family)	19.82
*Proteobacteria* phylum	19.42
*Coriobacteriia* class	19.22
*Bacilli* order	18.57

**Table 2:** Importance of the variables in model 3. This model was built including microbiome data (bacterial composition expressed as abundance) combined with dietary habits based on the Mediterranean diet compliance questionary analyzing each item separately to discern between individuals with CRC-related lesions (polyps or adenocarcinoma) versus individuals without pathological findings.

20 More Important Variables in the Model 3	*Importance*
Mediterranean diet item 3	100.00
Mediterranean diet item 7	54.90
*Sutterella* genus	26.30
*Oscillospirales* order	20.41
*Monoglobus* genus	15.77
*UCG-002* genus (*Oscillospiraceae* family)	15.76
*Proteobacteria* phylum	15.01
*Ruminococcaceae* family	14.64
*Monoglobales* order	13.65
*Streptococcaceae* family	13.64
*Streptococcus* genus	13.46
Mediterranean diet item 4	12.83
*Parasutterella* genus	12.49
*Eubacterium eligens* group	12.00
*Parabacteroides* genus	11.91
*Subdoligranulum* genus	11.24
*Burkholderiales* order	10.47
*Bacilli* class	10.35
*Monoglobaceae* family	10.30
*UCG-005* genus (*Oscillospiraceae* family)	10.18

## Data Availability

Collaborations with the CCR-microbiota Study are open to Biomedical Institutions, always after an accepted proposal for scientific work. Depending on the nature of the collaboration, electronic data, hard copy data, or biological samples should be provided. All collaborations will be made after a collaboration agreement. The terms of the collaboration agreement will be specific for each collaboration, and the extent of the shared documentation (i.e., deidentified participant data, data dictionary, biological samples, hard copy, or other specified data sets) will also be specifically established in light of each work.

## Supplementary Materials

The following supporting information can be downloaded at: https://www.mdpi.com/article/10.3390/nu17010084/s1, Figure S1: Confidence intervals for the ROC curves built based on the random forest classifier analysis.
