# Supplementary material for: Gut Microbiota Interacts with Dietary Habits in Screenings for Early Detection of Colorectal Cancer
Source: Nutrients. 2024 Dec 28;17(1):84. doi: 10.3390/nu17010084 (PMC11722828; doi:10.3390/nu17010084)
Supplement: Supplementary file 1 [file nutrients-17-00084-s001.zip › nutrients-3377477-supplementary.pdf]

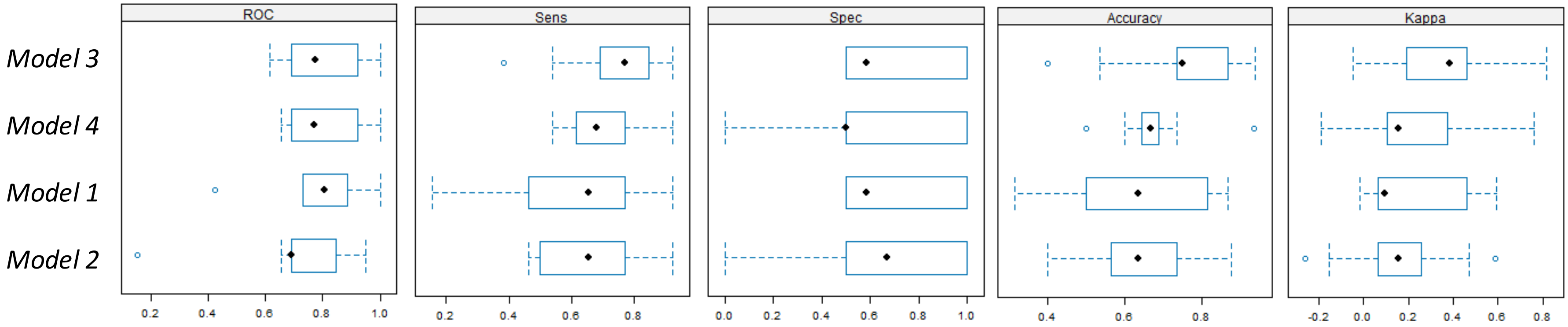

Dataset in the model	*ROC*	*Sensitibity*	*Specificity*	*Accuracy*	*Kappa*
1. Gut microbiota	0.790±0.160	0.629±0.225	0.717±0.249	0.648±0.187	0.240±0.238
2. Dietary habits in items	0.710±0.221	0.650±0.163	0.600±0.370	0.643±0.139	0.162±0.255
3. Gut microbiota + dietary habits (adherence)	0.801±0.138	0.737±0.167	0.717±0.249	0.736±0.160	0.345±0.264
4. Gut microbiota + dietary habits (items)	0.804±0.122	0.682±0.117	0.633±0.350	0.678±0.109	0.200±0.266

Figure S1. Confidence intervals for the ROC curves built based on the Random Forest Classifier analysis. Several random forest classifier models were created based on the following data: model 1) the microbiome (bacterial composition expressed as relative abundance); model 2) dietary habits based on the Mediterranean diet compliance questionary analyzing the 14 items together as a measure of the global adherence to the Mediterranean diet; model 3) the microbiome combined with dietary habits in term of adherence to a diet; model 4) the microbiome data combined with dietary habits based on the Mediterranean diet compliance questionary analyzing each item separately. In the table, values are mean ± standard deviation. In the graph, black dots represent median values.
